# BMC *Cardiovascular Disorders* reviewer acknowledgement 2015

**DOI:** 10.1186/s12872-016-0214-6

**Published:** 2016-02-15

**Authors:** Tim Shipley

**Affiliations:** BioMed Central, Floor 6, 236 Gray’s Inn Road, London, WC1X 8HB UK

## Abstract

The editors of BMC Cardiovascular Disorders would like to thank all our reviewers who have contributed to the journal in Volume 15 (2015).

**Flavia Ballocca**

Italy

**Matteo Bianco**

Italy

**Antonio H Frangieh**

Switzerland

**Franck Boccara**

France

**Sebastiano Gili**

Italy

**Karoly Kaszala**

USA

**Guido A. Matschuck Mhba**

Germany

**Robin Dullaart**

Netherlands

**John Hughes**

UK

**Toshinori Kamisako**

Japan

**Ryan Lennon**

USA

**Eiji Oda**

Japan

**Tony Merriman**

New Zealand

**Barry Palmer**

New Zealand

**Liang Chen**

China

**Andrea Chomistek**

USA

**Hannu Vanhanen**

Finland

**Umberto Benedetto**

UK

**Antonio Miceli**

Italy

**Susumu Manabe**

Japan

**Angelos Rigopoulos**

Germany

**Simone Biscaglia**

Italy

**Mario Iannaccone**

Italy

**Veronica D' Annunzio**

Argentina

**Liliana Nicolosi**

Argentina

**Dionyssios Leftheriotis**

Greece

**Vasilis Stasinos**

Greece

**Aldo Clerico**

Italy

**Armando Pucciarelli**

Italy

**Davide Giacomo Presutti**

Italy

**Giorgio Quadri**

Italy

**Fabrizio D'Ascenzo**

Italy

**Sven Meyer**

Germany

**Martina Zaninotto**

Italy

**Wayne Smith**

South Africa

**Julia Braun**

Switzerland

**Geetha Chittoor**

USA

**Francesca Viazzi**

Italy

**Hippokratis Kiaris**

Greece

**Jian Xiao**

China

**Brian Cooley**

USA

**Salma Esmaeil**

Egypt

**Rumi Faizer**

USA

**Eduardo Saadi**

Brazil

**Christian Delles**

UK

**Julian Walters**

UK

**Ignatios Ikonomidis**

Greece

**Agathi-Rosa Vrettou**

Greece

**Antonio Loforte**

Italy

**Maria Luisa Granada**

Spain

**Igor Koncar**

Serbia

**Emiliano Angeloni**

Italy

**Ana Olga Mocumbi**

Mozambique

**A Sampath Kumar**

India

**Guillermo Isasti**

Spain

**Raul Lopez-Salguero**

Spain

**María José Molina Mora**

Spain

**Ibrahim Akin**

Germany

**Michael Behnes**

Germany

**Stefano Pidello**

Italy

**Marta Bisi**

Italy

**Marco Pavani**

Italy

**Anna Grodzinsky**

USA

**Dany Jacob**

USA

**Carmelina Ariano**

Italy

**Imke Christiaans**

Netherlands

**Clemens Von Schacky**

Germany

**Jun Hu**

USA

**Linlin Li**

USA

**Guangliang Wang**

USA

**Abdulrahman Al-Malki**

Saudi Arabia

**Daniel Conklin**

USA

**Siyavash Joukar**

Iran

**Chiara Davico**

Italy

**Marianna Mazza**

Italy

**Alvaro Alonso**

USA

**Weihong Tang**

USA

**Jackie Boucher**

USA

**Ammar Ashor**

UK

**Henner Hanssen**

Switzerland

**Hsin-Fu Lin**

Taiwan

**Juan Gagliardi**

Argentina

**Manuel Rodriguez**

Argentina

**Alexander Berndt**

Germany

**Katja Grün**

Germany

**Isaac Aidonidis**

Greece

**Panagiota Flevari**

Greece

**Evangelos Repasos**

Greece

**Marijana Tadic**

Serbia

**Gianluca Campo**

Italy

**Yoichi Imori**

Switzerland

**Johannes Kragten**

Netherlands

**Asru Kumar Sinha**

India

**Sanjiv Dhingra**

Canada

**Ian Mcniece**

USA

**Tiina Heliö**

Finland

**Jelena Seferovic**

Serbia

**Zehra Bugra**

Turkey

**Vincent Robinson**

USA

**David Carty**

UK

**Carmel Mceniery**

UK

**David Freedman**

USA

**William Johnson**

UK

**Markus Juonala**

Finland

**Dario Celentani**

Italy

**Ping-Yen Liu**

Taiwan

**Vikram Agarwal**

USA

**Chirag Bavishi**

USA

**Parasuram Krishnamoorthy**

USA

**Kimberley Mellor**

New Zealand

**Michele Malaguarnera**

Italy

**Olga Mcleod**

Sweden

**Francesca Giordana**

Italy

**Gilles Lemesle**

France

**Marcus Franz**

Germany

**Thomas Meyer**

Germany

**Jeetesh Patel**

UK

**Peter Rahko**

USA

**Alistair Stewart**

New Zealand

**Madhulika Dixit**

India

**Yehuda Kamari**

Israel

**Jeffrey Brinker**

USA

**Ramez Nairooz**

USA

**Rita Pavasini**

Italy

**Michinari Nakamura**

USA

**Arintaya Phrommintikul**

Thailand

**Wilbert Aronow**

USA

**Bredy Pierre-Louis**

USA

**Devender Dhanda**

USA

**Giuseppina Di Biase**

Italy

**Marco Mennuni**

Italy

**Giulia Nangeroni**

Italy

**Lizhang Chen**

China

**Jingpu Shi**

China

**George Andrikopoulos**

Greece

**Dimitris Tsiachris**

Greece

**Fausto Biancari**

Finland

**Massimo Imazio**

Italy

**Na Li**

USA

**Michael Lipinski**

USA

**Michael Schwarzer**

Germany

**Lei Teng**

USA

**Yixuan Zhang**

USA

**Kurt Bestehorn**

Germany

**Michael Zairis**

Greece

**Barbro Kjellström**

Sweden

**Corey Tomczak**

Canada

**Amir Gahremanpour**

USA

**Yunn-Hwa Ma**

Taiwan

**Balázs Magyari**

Hungary

**Martina Pianelli**

Italy

**M.-Saadeh Suleiman**

UK

**Daniela Mazzaccaro**

Italy

**Giovanni Nano**

Italy

**Harry Smolen**

USA

**Shirling Lin**

Taiwan

**Jose Mill**

Brazil

**Charles Mondo**

Uganda

**Ayotunde Dokun**

USA

**Andrew Klein**

USA

**Naoyuki Takashima**

Japan

**Marek Brabec**

Republic Czech

**Christian Heiss**

Germany

**Antonis Vlassopoulos**

Switzerland

**Josef Dankiewicz**

Sweden

**Tinne Tranberg**

Denmark

**Sebastian Wiberg**

Denmark

**Oliver Gunkel**

Germany

**Mingyi Wang**

USA

**Vicky Cameron**

New Zealand

**Christopher Wong**

UK

**Spyridon Katsanos**

Greece

**Stamatios Lerakis**

USA

**Stylianos Orfanos**

Greece

**Nobuyuki Ohte**

Japan

**Danica Chen**

USA

**Augusto Montezano**

UK

**Ravi Sundaresan Nagalingam**

India

**Kai Lin**

USA

**Hajime Sakuma**

Japan

**Kyungeh An**

USA

**Wenqi Gan**

USA

**Peter Willeit**

UK

**Alessio Alogna**

Austria

**Heiner Post**

Germany

**Bronwyn Brown**

Australia

**Caroline Genco**

USA

**Mirela Habibovic**

Netherlands

**Kim Smolderen**

USA

**Kim Smolderen**

Netherlands

**Michael Stickland**

Canada

**Massimo Chello**

Italy

**Mario Petretta**

Italy

**Laura Perin**

USA

**Rajesh Ramasamy**

Malaysia

**Ntobeko Ntusi**

South Africa

**Welma Stonehouse**

Australia

**Welma Stonehouse**

New Zealand

**Jason Gill**

UK

**Raj Kumar Yadav**

India

**Branislava Ivanovic**

Serbia

**Sorina Mihaila**

Romania

**Grzegorz Bilo**

Italy

**Malgorzata Kloch-Badelek**

Poland

**Yu Jin**

Belgium

**Wiktoria Wojciechowska**

Poland

**Bo Hu**

USA

**Robert Biederman**

USA

**Saurabh Gupta**

India

**Ignacio Lugones**

Argentina

**Alanna Chamberlain**

USA

**Massimo Zoni-Berisso**

Italy

**Silvia Punzetti**

Italy

**Stefano Volpato**

Italy

**Thomas Gremmel**

Austria

**Konstantinos Katsanos**

Greece

**Paola Rizzo**

Italy

**Krisztina Heltai**

Hungary

**Lakshmi Mundkur**

India

**Emilio Vanoli**

Italy

**Tze-Fan Chao**

Taiwan

**Shao-Yuan Chuang**

Taiwan

**Chao-Yu Guo**

Taiwan

**Thomas Rosemann**

Switzerland

**Guillaume Lettre**

Switzerland

**Sebastien Theriault**

Canada

**Vasiliki Betihavas**

Australia

**David Schopfer**

USA

**Giacomo Boccuzzi**

Italy

**Alice Owen**

Australia

**David Preiss**

UK

**Li Jia**

China

**Yu Lu**

USA

**Amr Gad**

Egypt

**Giovanni Melina**

Italy

**Xiangwei Wu**

China

**Surendra Chutani**

India

**Decebal Gabriel Latcu**

Monaco

**Hassan Behlouli**

Canada

**Amresh Raina**

USA

**Dick Chan**

Australia

**Thomas Pulinilkunnil**

Canada

**Mahdi Najafi**

Iran

**Japhet Olisekodiaka**

Nigeria

**Hasan Shohag**

Australia

**Christoph Sinning**

Germany

**Miyuki Tsuchihashi-Makaya**

Japan

**Amber Courville**

USA

**Masakazu Hirata**

Japan

**Bernhard Maisch**

Germany

**Arsen Ristic**

Serbia

**Gurumurthy Hiremath**

USA

**Gen Suzuki**

USA

**Tadeusz Osadnik**

Poland

**Teresa Villarreal-Molina**

Mexico

**Ju Yi Chen**

Taiwan

**Pai Feng Hsu**

Taiwan

**Martin Schultz**

Australia

**Lin Xu**

Hong Kong

**Daniel Petrovic**

Slovenia

**Naoki Sato**

Japan

**Dmitry Shchekochikhin**

Russian Federation

**Torsten Hansen**

Germany

**Arti Shinde**

USA

**Xiaofang Lu**

USA

**Matthias Pauschinger**

Germany

**Luca Fileti**

Italy

**Arindam Maitra**

India

**Anna Lipowicz**

Poland

**Mads Nybo**

Denmark

**Francesco Perticone**

Italy

**Xin Tu**

China

**Robert Klautz**

Netherlands

**Yuqian Bao**

China

**Takeshi Matsumura**

Japan

**Julia Grapsa**

UK

**Kamal Mubarak**

USA

**Victoria Delgado**

Netherlands

**Knut Matre**

Norway

**Marjukka Kolehmainen**

Finland

**Giuseppe Ferrante**

Italy

**Gennaro Giustino**

USA

**Masahiro Natsuaki**

Japan

**Giuseppe Merla**

Italy

**David Buys**

USA

**Mizanur Rahman**

Bangladesh

**Bardia Arabkhani**

Netherlands

**Denise Baricocchi**

Italy

**Stjepan Jurisic**

Germany

**Emanuela Bostjancic**

Slovenia

**Yasuhide Kuwabara**

Japan

**Nina Zidar**

Slovenia

**Tobias Dünnwald**

Austria

**Yogish Kudva**

USA

**Erwan Leclair**

Canada

**Anup Karan**

India

**Luca Mascitelli**

Italy

**Tim Palmer**

UK

**John Hart**

USA

**Erik Holy**

Switzerland

**Patrick Mark**

UK

**Marius Miglinas**

Lithuania

**Naveed Akbar**

UK

**Louise Naylor**

Australia

**Pupalan Iyngkaran**

Australia

**Eric Loucks**

USA

**Paul Fedak**

Canada

**Katsuhito Fujiu**

Japan

**Frederic Jacques**

Canada

**Jiang Huai Wang**

Ireland

**Alida Caforio**

Italy

**Jitka Seidlerová**

Czech Republic

**Matteo Tebaldi**

Italy

**Jan Krejci**

Czech Republic

**Kelley Branch**

USA

**Muhib Khan**

USA

**Evan Thacker**

USA

**Abdallah Al-Mohammad**

UK

**Claudio Borghi**

Italy

**Giuseppe Giugliano**

Italy

**Regis Lamberts**

New Zealand

**Rainer G.H. Moosdorf**

Germany

**Natasa Bogavac-Stanojevic**

Serbia

**Nadia Hamdy**

Egypt

**Kim Connelly**

Canada

**David Playford**

Australia

**Jesse Dawson**

UK

**Moutaz Elkadri**

UK

**Charles Pearman**

UK

**Kerstin Hogg**

Canada

**Stephan Schirmer**

Germany

**Niels Van Royen**

Netherlands

**Manabu Hayakawa**

Japan

**Matteo Ghione**

Italy

**Fabio Mangiacapra**

Italy

**Michael Czubryt**

Canada

**Francesco Paneni**

Sweden

**Laura Burgers**

Netherlands

**Simone Huygens**

Netherlands

**Jian Xiong Chen**

USA

**Prasad Katakam**

USA

**Bin Wei**

USA

**Donal O'Gorman**

Ireland

**Georgios Theodorakis**

Greece

**Takumi Yamada**

USA

**Camille Lassale**

UK

**Yu-Guo Chen**

China

**Massimo Iacoviello**

Italy

**Marinella Tricarico**

Italy

**Sungjin Chung**

South Korea

**Tadashi Yoshida**

Japan

**Xiangjun Zeng**

China

**Wei Chen**

China

**Jennifer Strande**

USA

**Kirstine Calloe**

Denmark

**Douglas Curran-Everett**

USA

**Antony Jackson**

UK

**Morten Olesen**

Denmark

**Ander Regueiro Cueva**

Canada

**Majid Ghayour**

Iran

**Cyril Mamotte**

Australia

**Camilla Calvieri**

Italy

**Alfredo Rodriguez**

Argentina

**Amelia Carro**

Spain

**Martin Donato**

Argentina

**Federico Conrotto**

Italy

**Rami Doukky**

USA

**Venkatesh Murthy**

USA

**Randall Thompson**

USA

**Rozenn Quarck**

Belgium

**Mark Toshner**

UK

**Yoshihiro Fukumoto**

Japan

**Ana Savic-Radojevic**

Serbia

**Lei Wei**

USA

**Luigi Barberini**

Italy

**Trayambak Basak**

USA

**Sina Andalib**

Islamic Republic Of Iran

**Ricardo Perez De La Hoz**

Argentina

**Mehrdad Larry**

Islamic Republic Of Iran

**Aurelian Bidulescu**

USA

**Sayan Sen**

UK

**Susanne Unverzagt**

Germany

**Corstiaan Uil**

Netherlands

**Michael Bursztyn**

Israel

**Megan Wenner**

USA

**Mackram Eleid**

USA

**Oluseun O. Alli**

USA

**Hongyu Wang**

China

**Ichiro Tatsuno**

Japan

**Florian Rader**

USA

**Frangiscos Parthenakis**

Greece

**Angel Sanchez-Recalde**

Spain

**Salah Said**

Netherlands

**Jamilah Al Rahimi**

Saudi Arabia

**Ali Shafiq**

USA

**Matthew Jankowich**

USA

**S Andreas**

Germany

**Imran Iftikhar**

USA

**Pablo Salinas**

Spain

**Sang Hong Baek**

Democratic People'S Republic Of Korea

**Shozo Sueda**

Japan

**Stavros Tzortzis**

Greece

**Alexandra Frogoudaki**

Greece

**Jeffrey A. Brinker**

USA

**Masanori Taniwaki**

Japan

**Alessio Mattesini**

Italy

**Bledar Daka**

Sweden

**Sukhjinder Nijjer**

UK

**David Vivas**

Spain

**Jan Vojacek**

Czech Republic

**Robert Bauer**

USA

**Marijana Tadic Serbia And**

Montenegro

**Marcio Kiuchi**

Brazil

**Takeshi Takami**

Japan

**Neal Benowitz**

USA

**Jamie Hartmann-Boyce**

UK

**Agathi Vrettou**

Greece

**Spiridon Katsanos**

Greece

**Gianluca Faggioli**

Italy

**P De Rango**

Italy

**Muhib Khan**

USA

**Zhu-Ming Zhang**

USA

**Yuichiro Yano**

USA

**Inmaculada Moreno**

USA

**José Manuel García Pinilla**

USA

**Noela Rodríguez-Losada**

USA

**Alberto Crottogini**

Argentina

**Jai Singh**

UK

**Szu-Yuan Li**

Taiwan, Republic Of China

**Szu-Chia Chen**

Taiwan, Republic Of China

**Thanh Phan**

Australia

**Alfonso Ielasi**

Italy

**Stella Stabouli**

Greece

**Sanaz Sedaghat**

Netherlands

**Ahmet Karabulut**

Turkey

**Salvatore Carbone**

Italy

**Rebecca Levitt**

USA

**Kelvin Wong**

Australia

**Yunlong Huo**

China

**Ellen Freel**

UK

**Tatiana Kuznetsova**

Belgium

**Melinda Carrington**

Australia

**Adelaide Fusco**

Italy

**Masao Saotome**

Japan

**Eric Lopatta**

Germany

**James Moon**

UK

**João Cavalcante**

USA

**Luca Di Lullo**

Italy

**Seiji Umemoto**

Japan

**Ryan Lennon**

USA

**Douglas Curran-Everett**

USA

**Sebastian Garcia Zamora**

Argentina

**Alexander Fuernkranz**

Germany

**Zachary Bloomgarden**

USA

**Zdenek Turek**

Czech Republic

**Hynek Riha**

Czech Republic

**Salvatore Di Stefano**

Spain

**Emiliano Rodríguez-Caulo**

Spain

**Suzanne Van Dijk**

Netherlands

**Tatjana Rundek**

USA

**Warren Gasper**

USA

**Mark Koelemay**

Netherlands

**Sammy Chan**

Canada

**Prem Shekar**

USA

**Carlos Porras**

Spain

**Kim Smolderen**

USA

**Agnieszka Kapłon-Cieślicka**

Poland

**Una Martin**

UK

**Cornelia Deutsch**

Germany

**Sreek Vemulapalli**

USA

**Massimo Fineschi**

Italy

**Wayne L. Miller**

USA

**George Theodorakis**

Greece

**Yasihiro Yoshiga**

Japan

**Flavia Del Porto**

Italy

**Cecilia Hertig**

Argentina

**Mustafa Cikirikcioglu**

Switzerland

**Olivier Bouchot**

France

**Stelios Tzeis**

Greece

**Fred Buckhold**

USA

**Andreas Schneider**

Germany

**S E Kjeldsen**

Norway

**Sante Pierdomenico**

Italy

**Iddo Ben-Dov**

Israel

**D Tsiapras**

Greece

**George Makavos**

Greece

**Tain-Yen Hsia**

UK

**Gemma Sánchez-Espín**

Spain

**Carlos Velazquez**

Spain

**Virginia Miller**

USA

**Ahmet Can**

Turkey

**Giampaolo Morciano**

Italy

**Fausto Rigo**

Italy

**Suzanne Fredericks**

Canada

**Jacek Bil**

Poland

**Demos Katritsis**

Greece

**Xenophon Costeas**

Greece

**Sushmita Parashar**

USA

**Rosa Sicari**

Italy

**Elizabeth Cross**

UK

**Sanjay Chatterjee**

India

**Chirag Bavishi**

USA

**Tudor Pörner**

Germany

**Yves St-Pierre**

Canada

**Manlio Marquez**

Mexico

**Robert Scragg**

New Zealand

**Takafumi Okura**

Japan

**Sumusu Ogawa**

Japan

**Tomasz Guzik**

UK

**Senbeta Abdissa**

Ethiopia

**Zsolt Bagyura**

Hungary

**Mohamed Abdel Rahman**

Egypt

**John Saxon**

USA

**Delphine De Smedt**

Belgium

**Yumei Gu**

Belgium

**Johann Auer**

Austria

**Stylianos Kapiotis**

Austria

**Jens Jordan**

Germany

**Dorota Drozdz**

Poland

**Janne Maier**

USA

**Catherine Bagot**

UK

**Stefania Battista**

Italy

**Roberto Gambino**

Italy

**Brian Graham**

USA

**Solomon Abebe**

Ethiopia

**A.K.M. Islam**

Bangladesh

**K.M.H.S. Haque**

Bangladesh

**Michael Toth**

USA

**Carlo Barbetta**

Italy

**Alison Short**

Australia

**Joanne Loewy**

USA

**Kun-Ta Chou**

Taiwan

**Fatima Cintra**

Brazil

**Neil Heron**

UK

**Rohan Khera**

USA

**Agnieszka Olszanecka**

Poland

**Amenah Jaghoori**

Australia

**Tetsuo Sasano**

Japan

**Wai-Meng Kwok**

USA

**Alexandros Briasoulis**

USA

**Mehmet Birhan Yilmaz**

Turkey

**Emmy Okello**

Uganda

**Markus Ferrari**

Germany

**Jiunn-Lee Lin**

Taiwan

**T Leiria**

Brazil

**Gil Salles**

Brazil

**Andrea Schrepper**

Germany

**Renzo Levi**

Italy

**Peter Weeke**

Denmark

**Christina Werner**

Germany

**Seong-Kyu Lee**

Republic Of Korea

**Jeffrey Breall**

USA

**Oriol Rebagliato Nadal**

Spain

**Andrew Don-Wauchope**

Canada

**Panagiota Christia**

USA

**Antonio Frangieh**

Switzerland

**Laurence G. Howes**

Australia

**Ju-Yi Chen**

Taiwan

**Ales Linhart**

Czech Republic

**Mariano Pellicano**

Belgium

**Themistoklis Maounis**

Greece

**John Palios**

Greece

**Liwen Li**

China

**Emmanouil Kanoupakis**

Greece

**Anne Child**

UK

**Cristiana Catena**

Italy

**Gavin Norton**

South Africa

**Muharrem Akin**

Germany

**Celestino Sardu**

Italy

